# Bladder Cancer Management in Brazil's Supplementary Health System: Real-World Practice Patterns

**DOI:** 10.1590/S1677-5538.IBJU.2026.0218

**Published:** 2026-07-20

**Authors:** Isabella Freitas Astua, Caio Vinícius Suartz, Daher C. Chade, Daniel de Freitas Gomes Soares, Augusto Modesto de Souza, Roni de Carvalho Fernandes, Fernando Korkes

**Affiliations:** 1 Faculdade de Medicina do ABC - FMABC Santo André SP Brasil Faculdade de Medicina do ABC - FMABC, Santo André, SP, Brasil; 2 Northern Ontario School of Medicine Urology Department Thunder Bay Canada Northern Ontario School of Medicine, Urology Department, Thunder Bay, Ontario, Canada; 3 Instituto do Câncer do Estado de São Paulo São Paulo SP Brasil Instituto do Câncer do Estado de São Paulo (ICESP), São Paulo, SP, Brasil; 4 Universidade de São Paulo São Paulo Brasil Universidade de São Paulo - USP, São Paulo, Brasil; 5 Hospital Beneficência Portuguesa de São Paulo São Paulo Brasil Hospital Beneficência Portuguesa de São Paulo, São Paulo, Brasil; 6 Santa Casa de Porto Alegre Porto Alegre RS Brasil Santa Casa de Porto Alegre, Porto Alegre, RS, Brasil; 7 Hospital Aristides Maltez Salvador BA Brasil Hospital Aristides Maltez, Salvador, BA, Brasil; 8 Faculdade de Ciências Médicas da Santa Casa de São Paulo São Paulo SP Brasil Faculdade de Ciências Médicas da Santa Casa de São Paulo, São Paulo, SP, Brasil; 9 Hospital Israelita Albert Einstein São Paulo SP Brasil Hospital Israelita Albert Einstein, São Paulo, SP, Brasil

**Keywords:** Urinary Bladder Neoplasms, Urologists, Delivery of Health Care

## Abstract

**Purpose:**

The objective of the present study was to evaluate real-world practices of Brazilian urologists regarding bladder cancer management within the supplementary healthcare system.

**Materials and Methods:**

A cross-sectional, survey-based study was conducted among Brazilian urologists. A structured questionnaire assessed demographics, practice characteristics, and management strategies for non–muscle-invasive (NMIBC) and muscle-invasive bladder cancer (MIBC). Data were collected during the 2025 Brazilian Congress of Urology and via electronic distribution. Descriptive statistics were used, and exploratory comparisons were performed.

**Results:**

A total of 914 urologists from 25 federative units (24 states and the federal district) participated in the study. Cystoscopy was nearly universal (93.8%), but access to enhanced imaging (17.9%) and office-based flexible cystoscopy (19.3%) was limited. In NMIBC, most urologists maintained patient care (82.9%), but management was heterogeneous. Immediate postoperative intravesical chemotherapy was rarely used. Bacillus Calmette-Guérin (BCG) was prescribed in most cases of recently treated NMIBC by 39.3% of respondents, while 54.2% referred patients for administration. Although 74.5% reported access to it, only 14.3% had availability for all patients. Preference flow analysis showed BCG as the main first-line therapy (65.4%), intravesical chemotherapy as second-line (51.2%), and pembrolizumab (31.3%) and radical cystectomy (30.2%) as third-line options. Radical cystectomy is annually performed by 65.1% of respondents; however, most reported low annual volumes, with 95% performing fewer than ten cases per year.

**Conclusions:**

Bladder cancer management in the Brazilian supplementary healthcare system is heterogeneous, with important gaps in access, technology availability, and adherence to recommended practices. Despite limitations inherited to survey-based research, including potential selection bias, reliance on self-reported data, and possible overlap between public and private practice settings, these findings highlight opportunities for educational initiatives and system-level improvements.

## INTRODUCTION

Brazil has a complex healthcare structure composed of a universal public healthcare system (SUS), and a large supplementary private healthcare sector that covers approximately one quarter of the population. While SUS provides universal access to medical care, the supplementary health system offers private insurance-based coverage that often provides broader access to diagnostic technologies and advanced treatments (1, 2). More recently adopted treatments for bladder cancer, such as immunotherapy or antibody-drug conjugates, are only available in the private system. Differences between these two systems can influence physician practice patterns, access to therapies, and the organization of oncological care.

Bladder cancer represents a clinically relevant disease due to its high recurrence rate, the need for long-term surveillance, high mortality, and financial impact (3). Management of non–muscle invasive bladder cancer (NMIBC) and muscle-invasive bladder cancer (MIBC) requires access to diagnostic procedures such as cystoscopy, transurethral resection of bladder tumors (TURBT), intravesical chemotherapy, and complex surgical procedures, including radical cystectomy (RC). The availability of these resources and reimbursement models may vary considerably within the supplementary healthcare system (4).

Brazil has one of the largest urology communities in the world, with around 6,600 practicing urologists distributed across the country (5). It also represents one of the largest markets for the pharmaceutical industry. Understanding how these specialists diagnose and treat bladder cancer within the private healthcare environment is important for identifying barriers, technological gaps, and variations in treatment strategies. We hypothesized that there is substantial heterogeneity in the real-world management of bladder cancer in the Brazilian supplementary healthcare system.

The objective of the present study was to evaluate the real-world practices of Brazilian urologists regarding bladder cancer management within this setting.

## MATERIALS AND METHODS

This study was designed as a cross-sectional, descriptive, survey-based study aimed at evaluating the real-world clinical practice patterns of Brazilian urologists regarding the management of bladder cancer in the supplementary (private) healthcare setting. The primary objective was to characterize diagnostic and therapeutic strategies used in routine practice, with particular emphasis on the treatment of non–muscle-invasive bladder cancer and muscle-invasive bladder cancer within the Brazilian private healthcare system.

The target population consisted of practicing urologists in Brazil. Based on data from the Federal Medicine Council (CFM), the total population of active urologists in Brazil is approximately 6,600 at the time of study design (5). Eligible participants included board-certified urologists and urology practitioners from all geographic regions of the country, regardless of years in practice or institution affiliation, who were involved in bladder cancer care in the private setting.

A structured questionnaire was developed by the study investigators based on clinically relevant aspects of bladder cancer management in Brazil, focusing on areas where practice variation is expected due to differences in access, reimbursement, institutional protocols, and the adoption of new technologies. The questionnaire included items related to physician demographics, geographic region, practice setting, years in practice, number of bladder cancer patients, and therapeutic preferences in specific clinical scenarios. Questions addressed diagnostic work-up, intravesical therapy, management of BCG-unresponsive disease, perioperative strategies, referral patterns for radical cystectomy, use of systemic therapy, and access to newer treatments in the supplementary healthcare setting. The questionnaire was not formally validated. However, it underwent pilot-testing with five urologists who completed the survey and provided feedback regarding question clarity, comprehensiveness, and relevance to clinical practice. Minor adjustments were made based on their suggestions before final distribution.

To ensure adequate representativeness of bladder cancer management practices among Brazilian urologists, the sample size was calculated based on an estimated population of 6,600 active urologists in Brazil (5). A 95% confidence level, 5% margin of error, and 50% response distribution were assumed; the latter was chosen to provide the most conservative estimate. The sample size for an infinite population was first calculated using the standard formula for proportions and subsequently adjusted using the finite population correction. Under these assumptions, the minimum required sample size was 364 respondents. To account for potential nonresponse and incomplete questionnaires, the survey sought to exceed this threshold, thereby enhancing the reliability of the findings and enabling exploratory subgroup analyses across different geographic regions and practice settings.

Data were collected during the Brazilian Congress of Urology in 2025 through a structured questionnaire. The survey was administered by ten trained interviewers, who invited attending urologists to participate in the study. Although the exact number of urologists approached in person was not recorded, according to the reports from the Brazilian Society of Urology (SBU), the congress had more than 3,000 participants. A total of 604 responses were obtained during the event. In addition to in-person recruitment, the questionnaire was distributed electronically via email and WhatsApp using existing databases of Brazilian urologists. A total of 4,397 urologists received the electronic invitation, of whom 1,115 opened the message and 310 completed the questionnaire. Participants were asked to voluntarily complete the survey and the responses were collected anonymously, while ensuring that each participant completed the survey only once. All responses were compiled into a centralized database for descriptive statistical analysis.

All questionnaire items were configured as mandatory; therefore, no missing data occurred in the conventional sense, and all 914 participants completed the survey in full. However, for questions that included an open-ended response option ("Other"), a small number of responses were excluded from analysis due to lack of meaningful content (e.g., isolated characters or non-interpretable symbols such as "." or "-"). These exclusions were treated as part of data cleaning rather than missing data. The number of excluded responses was low across variables: years since graduation (n=3), state of practice (n=7), number of bladder cancer patients treated per year (n=6), number of cystectomies per year (n=7), practice in the public health system (n=7), and clinical management scenarios for NMIBC and MIBC (n=8 each), corresponding to less than 1% of responses per item. No formal assessment of missing data mechanisms was performed, as no item-level missingness was present. Given the minimal proportion and apparently non-systematic nature of these exclusions, the risk of bias is likely negligible.

Participation in the survey was voluntary, anonymous, and without financial compensation. No personally identifiable information was collected. Because the study involved anonymized physician-reported practice patterns and did not include patient-level data, the risk to participants was considered minimal. The study was conducted in accordance with applicable ethical standards for survey-based research. Under Article 1, sole paragraph, items I and VII, of Brazilian National Health Council Resolution No. 510/2016, this study was exempt from registration and ethical review by the CEP/CONEP system, as it consisted of an anonymous public opinion survey and an analysis of situations derived from professional practice, with no identifiable participant data.

Descriptive statistics were used to summarize respondent characteristics and survey responses. Categorical variables were presented as absolute and relative frequencies (numbers and percentages). Associations between categorical variables were evaluated using the chi-square test or Fisher's exact test, as appropriate according to sample distribution. Subgroup analyses were performed to compare practice patterns according to geographic region and practice-related variables. A significance level of 95% was adopted, and p-values < 0.05 were considered statistically significant. Statistical analyses were performed using Stata version 18.0. Missing data was handled by available-case analysis, and percentages for individual items were calculated based on the number of respondents who completed each specific question.

## RESULTS

### Demographic aspects

A total of 914 urologists completed the survey, corresponding to 13.6% of the 6,690 urologists practicing in Brazil, with responses obtained from 25 federative units. State-level representativeness ranged from 8.6% to 48.5%. The mean experience with urological practice among respondents was 17.7 ± 8.8 years (range: 0–57 years).

Among respondents, 85.1% reported working in private practice, frequently in association with major insurance providers, while nearly three-quarters also reported working within the public health system (SUS) (72.7%).

Many respondents rated reimbursement for cystoscopy, TURBT and radical cystectomy as poor or very poor, reaching 81.5% for cystoscopy.

### Initial diagnosis and management of bladder cancer

Cystoscopy was widely adopted, being routinely performed by nearly all respondents (93.8%), with more than half reporting office-based cystoscopy. However, access to advanced technologies such as flexible cystoscopy and narrow-band imaging remained limited (Table-1).

**Table 1:** Cystoscopy and TURBT practice patterns among Brazilian urologists.

Variable	n (%)
Cystoscopy setting ^a^	
Operating room cystoscopy	857 (93.8)
Office-based cystoscopy	508 (55.6)
Flexible cystoscopy	176 (19.3)
Narrow-band imaging	164 (17.9)
TURBT surgical practice	
Most used energy source	
Monopolar	381 (41.7)
Bipolar	513 (56.1)
Laser	18 (2.0)
Preferred technique	
Conventional TURBT	513 (56.1)
En bloc TURBT	105 (11.5)
Both techniques	286 (33.1)
re-TURBT performance	
Always	230 (25.2)
Most cases	631 (69.0)
Rarely	44 (4.8)
Never	9 (1.0)

Note: TURBT = transurethral resection of bladder tumor a Multiple choices were allowed for the cystoscopy setting question.

Office-based cystoscopy was more frequently performed among urologists who reported adequate or good reimbursement compared to those reporting poor reimbursement (72.9% vs. 53.0%). This difference was statistically significant (p < 0.001).

Most urologists reported a low annual volume of bladder cancer cases, with 52.1% treating between one and five patients per year. Despite this, hematuria evaluation was nearly universal in clinical practice (99.5%). The diagnostic approach varied, with urinalysis, ultrasound, computed tomography and cystoscopy frequently used, whereas advanced imaging modalities were uncommon.

When imaging suggested bladder tumor, most urologists preferred to combine diagnostic cystoscopy with immediate TURBT (84%). Bipolar resection was the most commonly used technique, whereas conventional TURBT remained the predominant surgical approach (Table-1).

Immediate postoperative intravesical chemotherapy was infrequently used, with most urologists reporting that they never prescribe it (65%). No statistically significant association was observed between the use of postoperative intravesical chemotherapy and geographic region of practice (all p > 0.05).

Following NMIBC diagnosis, most urologists continued managing patients themselves (82.9%), with a minority referring patients to clinical oncologists or other specialists.

### Continued treatment, more complex NMIBC follow-up and intravesical treatments

Second TURBT was commonly performed, although not universally adopted, with 69% reporting its use in most cases and 25.2% in all cases. Management of intravesical chemotherapy showed substantial variability, with many urologists delegating treatment definition and/or administration to clinical oncologists.

For patients with NMIBC who had previously received treatment, most urologists reported not prescribing BCG and instead referring patients to a clinical oncologist. Conversely, 39.3% prescribed BCG in the majority of cases (Table-2).

**Table 2:** BCG availability and prescribing patterns among Brazilian urologists.

Variable	n (%)
BCG availability	
Available for all	131 (14.3)
Available for the vast majority	324 (35.4)
Available, but just for some	226 (24.7)
Not available	188 (20.6)
Unknown	45 (4.9)
BCG after HR-NMIBC a	
Prescribe BCG	359 (39.3)
Refer the patient to an urologic oncologist for prescription	60 (6.6)
Refer the patient to a clinical oncologist for prescription	495 (54.2)
‘BCG protocol	
6-week induction + 3x maintenance (1–3 years) – SWOG b	443 (48.5)
6-week induction + monthly maintenance (1–3 years)	286 (31.3)
Other protocol	13 (1.4)
No regular BCG administration	67 (7.3)
Unknown	105 (11.5)

Note: BCG = Bacillus Calmette-Guérin. a HR-NMIBC = high-risk non-muscle-invasive bladder cancer. b SWOG = Southwest Oncology Group.

BCG use was heterogenous. Although access was reported by most respondents, consistent availability for all patients was limited, and a considerable proportion reported no access. Among the respondents, the SWOG BCG regimen, consisting of a 6-week induction course followed by maintenance therapy for 1–3 years, was the most frequently adopted protocol (Table-2).

BCG prescription patterns were not significantly associated with the degree of access to BCG (p = 0.353), as referral to other specialists remained common even among respondents reporting broad access to the drug. However, significant regional differences were observed regarding BCG prescription patterns. Urologists practicing in the Northeast (p = 0.011), Southeast (p < 0.001), and South (p = 0.012) were more likely to prescribe BCG directly, whereas referral to another specialist was more frequent in other regions.

In cases of BCG unresponsive disease, the most commonly selected management options were intravesical chemotherapy (43.1%), radical cystectomy (41.5%), repeat TURBT alone (27%), pembrolizumab (25.4%), and repeat BCG (18.6%). Multiple answers were allowed for this question.

In a hypothetically healthy patient diagnosed with NMIBC, BCG remained the preferred first-line therapy (86%), followed by intravesical chemotherapy and, less frequently, pembrolizumab and radical cystectomy. Treatment sequencing analysis confirmed BCG as the most common first-line option (65.4%), with intravesical chemotherapy predominating in second-line settings and systemic or surgical approaches more frequently selected in later lines of treatment (Figure-1).

**Figure 1:**
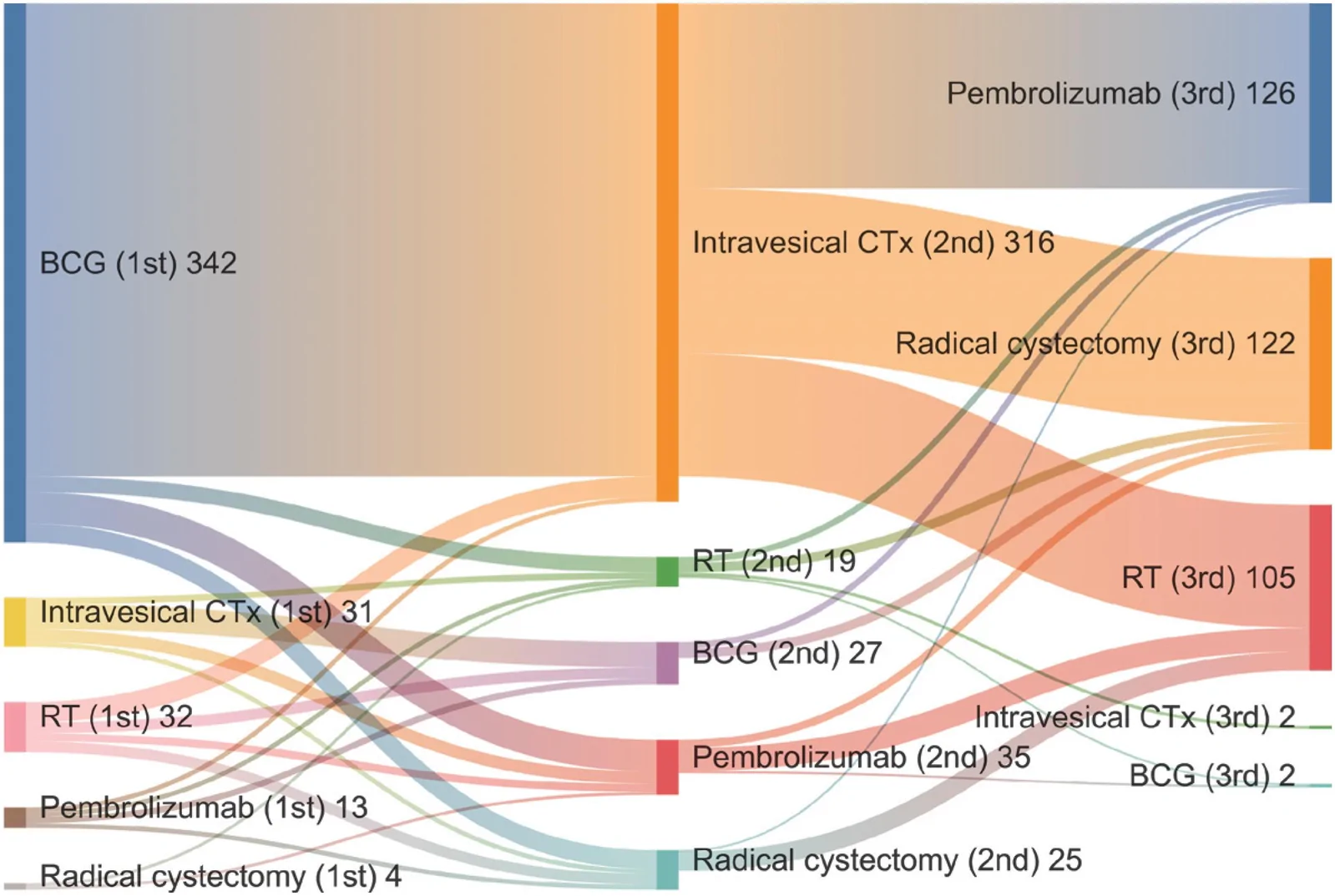
Sankey diagram of the most common sequential treatments of HR-NMIBC according to 914 respondent urologists. The diagram illustrates the flow of treatment preferences across first-, second-, and third-line options, with stream width proportional to the number of respondents.

It shows how initial choices distribute into subsequent treatment options, allowing visualization of the most common therapeutic sequences.

Intravesical CTx = Intravesical Chemotherapy; RT = Radiotherapy

A substantial proportion of respondents reported not prescribing intravesical chemotherapy (42.9%). Among those who did, referral for administration was common (64.5%). Gemcitabine was the most frequently used agent (44.1%), followed by mitomycin C and combination regimens. Also, 46.0% reported prescribing none of these agents.

Regarding emerging therapies for NMIBC, systemic immunotherapy was perceived as the most promising approach (56.5%), followed by TAR (29.9%), BCG (18.7%), intravesical chemotherapy (18.7%), Adstiladrin (12.9%), Anktiva (8.4%), Cretostimogene Grenadenorepvec (5.8%-), and Oncotehrad (4.2%). However, BCG and intravesical chemotherapy were considered the most clinically relevant therapies in current practice.

### MIBC treatment and radical cystectomy practices

Following MIBC diagnosis after TURBT, most urologists continued patient management (65.3%), although referral to specialized urologists or clinical oncologists was also common.

Radical cystectomy was reported to have been performed by 703 respondents. Of these, 91.5% performed the procedure themselves, while 8.5% invited a more experienced surgeon to assist. Referral to specialized centers was reported by 20.1%.

RC is annually performed by 65.1% of respondents, however, surgical volume was generally low. Within the last year, 36% did not perform any surgeries, and most urologists reported fewer than five procedures per year (77.7%).

Open surgery was the most performed exclusive approach (28.5%), although minimally invasive and robotic techniques were also reported. Ileal conduit was the most frequently performed urinary diversion (73.0%), while a notable proportion of respondents reported not performing any type of diversion (21.1%) (Figure-2).

**Figure 2:**
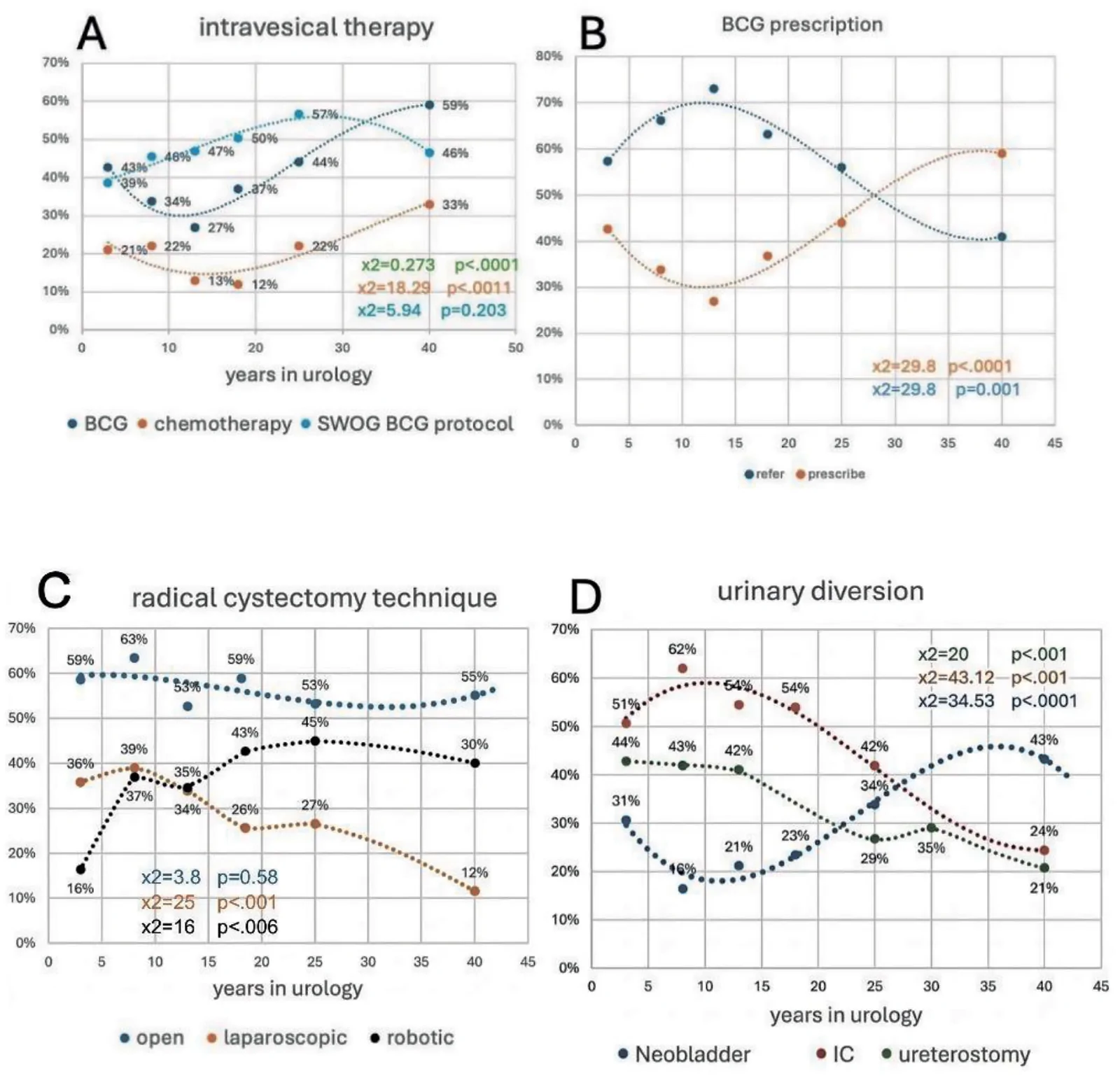
Practice patterns according to years of urological experience.

A) Intravesical therapies prescribed and BCG protocol. B) referral or prescription of intravesical BCG. C) radical cystectomy techniques.

D) common techniques of urinary diversion.

Most urologists referred patients to clinical oncologists for neoadjuvant therapy rather than managing it directly (79.4%).

Finally, bladder-preserving strategies such as radiotherapy or intravesical chemotherapy were generally considered second-line options, typically reserved for selected cases (60.9%).

## DISCUSSION

The incidence of bladder cancer in Brazil has risen during the last decade (6). Most procedures to treat bladder cancer are performed by community urologists, and there is a paucity of information on how these patients are treated. In recent years, there have been dramatic changes in technologies and medications available to treat both NMIBC and MIBC. Several factors, such as cultural differences, basic training, structure, and medical knowledge, may play a major role in the heterogeneity of care. Understanding these gaps may help guide educational strategies, resource allocation, and quality improvement initiatives tailored to local needs.

Our findings suggest that cystoscopy is widely incorporated into urological practice in Brazil, reinforcing its role as a fundamental diagnostic tool. However, the pattern of use highlights structural and technological limitations within the healthcare system. The predominance of rigid cystoscopy in office-based settings, along with the limited availability of flexible cystoscopes and enhanced visualization techniques, suggests important limitations in access to contemporary diagnostic technologies (Table-1).

Financial factors appear to play a significant role in shaping this scenario. The overall perception of inadequate reimbursement for cystoscopy, together with its association with lower adoption of office-based procedures, suggests that economic constraints may directly influence clinical decision-making. In this context, suboptimal reimbursement may act as a barrier not only to the incorporation of more efficient technologies, but also to adherence to recommended surveillance strategies in NMIBC patients. These findings likely reflect broader systemic issues, including resource limitations, healthcare organization, and a lower degree of subspecialization and referral compared to higher-income settings. A recent nationwide analysis of bladder cancer procedures within the Brazilian public health system demonstrated significant regional disparities in surgical volume, mortality, and access to care, reinforcing the presence of structural inequalities that may directly impact treatment delivery (7).

Consistent with international trends, bipolar TURBT has gained widespread acceptance (8), and represents the technique mostly used by Brazilian urologists. Additionally, en bloc resection has become more commonly debated in recent years (8). In our survey, we have noticed that almost half of the urologists have been applying this technique. Conventional TURBT, however, remains the most used technique in most cases (Table-1).

Single instillation of an intravesical chemotherapy agent after a conventional low/intermediate-risk TURBT is a recommended practice. However, only 10.1% of Brazilian urologists consistently practice this. For the vast majority, this practice is never performed (Table-3). This is a point that should be addressed through educational initiatives, since this practice is associated with up to 12% decrease in NMIBC recurrence (9). It is important to reinforce, however, that this practice is very difficult to be accepted by health insurance in Brazil, and there are logistical challenges to overcome this current limitation.

**Table 3:** Reported Brazilian Practice Patterns Compared with Guideline Recommendations.

Topic	Brazilian Practice	SBU (2010)	AUA (2024)	NCCN (2026)	EAU (2026)
Immediate Intravesical Chemotherapy	65% of urologists report they never prescribe it	Instillation within 6 hours of TURBT	For low-risk and small papillary Ta recurrences (> 1 year after previous TURBT) within 24 hours	Single dose within 24 hours	For low-risk cases within 24 hours
Re-TURBT	69% perform it in most cases; 25.2% perform it in all cases	For incomplete/high-volume tumors, T1, or if muscle is absent from specimen	For incomplete resections, high-grade Ta, or if muscle is absent. Timing: 2–6 weeks	For high-risk, incomplete visually resections, high-grade, or if muscle is absent	Standard practice for T1 high-grade within 6 weeks
BCG for High-Risk NMIBC	86% of specialists choose BCG as initial therapy	Superior to chemotherapy for reducing progression	Full-dose induction followed by maintenance BCG for reducing progression to invasive disease	Selected as first-line BCG for induction and maintenance phases	BCG induction and maintenance phases
BCG Maintenance	Most follow SWOG protocol (6-week induction course followed by maintenance therapy for 1-3 years)	At least one year of maintenance is necessary	Recommended for three years in high-risk patients to prevent recurrence	One year for intermediate-risk and three years for high-risk patients	Ideally one year for intermediate; one to three years for high and very high-risk
Management after BCG Failure	Intravesical chemo (43%), Cystectomy (41.5%), Pembrolizumab (25.4%), Repeat BCG (18.6%)	Early radical cystectomy is recommended for all failure within 6 months	Preferred: Radical Cystectomy. Bladder-sparing: Pembrolizumab, TAR-200, Nadofaragene, or N-803 + BCG	Preferred: Radical Cystectomy. Bladder-sparing: Nadofaragene, N-803+BCG, or Pembrolizumab	Primary: Offer radical cystectomy. Alternatives: Nadofaragene, Gem/Doc, or Pembrolizumab (for CIS)

Source: Elaborated by the authors

These findings are consistent with previous national data. A prior Brazilian survey also demonstrated low adherence to guideline-recommended immediate intravesical chemotherapy in low-risk disease, with substantial variability in clinical practice patterns. Despite the time gap between studies and major advances in NMIBC landscape including bladder-preserving approaches such as gene therapy, immune checkpoint inhibitors, and IL-15 superagonists for BCG-unresponsive disease, adherence to immediate postoperative intravesical chemotherapy in Brazil remains consistently low. Considering that this recommendation has been consistently endorsed by international guidelines for more than a decade, our findings suggest that barriers to guideline implementation remain largely unresolved (10-14).

Another recommended practice for most cases is to repeat a TURBT (Re-TURBT), mostly after a T1 or high-grade disease, if the muscle was not represented. For the vast majority of urologists, Re-TURBT is commonly performed. This is an important good practice, as it is associated with an additional 50% risk of residual NMIBC and an 8% risk of up-staging (15).

Although our manuscript highlights variability in clinical practice, a formal comparison with contemporary international guidelines (EAU, AUA, and NCCN) provides additional perspective. Adherence appeared heterogeneous across different domains of NMIBC management. High concordance was observed for practices such as repeat TURBT in high-risk disease, whereas substantial gaps were identified in the use of immediate postoperative intravesical chemotherapy and maintenance BCG therapy. These findings suggest that, although several key recommendations have been successfully incorporated into clinical practice, others remain underutilized, particularly in settings affected by logistical, economic, and resource-related constraints (11-14) (Table-3). This interpretation is supported by recent Latin American multicenter data demonstrating frequent delays in BCG initiation and low adherence to maintenance schedules across the region (16).

Brazilian guidelines for NMIBC, published under the supervision of the Sociedade Brasileira de Urologia (SBU), are largely based on recommendations from the European Association of Urology (EAU) and demonstrate substantial concordance with contemporary quality control/performance indicators (QCIs/QPIs) for bladder cancer management (14). Internationally recognized quality metrics consistently include adequate TURBT with detrusor muscle sampling, appropriate use of repeat TURBT in high-risk disease, immediate postoperative intravesical chemotherapy, and standardized surgical documentation (17, 18). In this context, Brazilian recommendations remain broadly aligned with internationally adopted quality indicators associated with improved staging accuracy, treatment adequacy, and oncological outcomes (17, 19). Although current QCIs place increasing emphasis on operative reporting and documentation quality, including tumor characteristics, completeness of resection, and intravesical therapy administration, the overall principles remain consistent with current Brazilian guideline recommendations (14, 19, 20).

As previously mentioned, a relevant finding from the present study is the apparent shift in the management of high-risk NMIBC (HR-NMIBC), with many urologists no longer directly prescribing or administering intravesical BCG despite its well-established role as the standard of care following adequate TURBT (and Re-TURBT for most patients) (Table-2). Although BCG has historically been managed within urology practice since the 1980s (13), our data suggest an increasing tendency to refer these patients to medical oncologists or other specialists. Notably, the lack of association between BCG availability and the referral patterns reinforces the hypothesis that factors beyond simple access are involved. Elements such as referral pathways, institutional workflows, increasing subspecialization, and logistical challenges related to BCG administration likely play a central role in shaping care delivery. In addition, the prolonged shortage of BCG in Brazil over the past decade (21) may have contributed to a loss of familiarity or routine among some urologists, further supporting this apparent shift in practice. This is consistent with the observed variation according to years of practice, with higher referral rates among urologists with five to 18 years of practice.

This observed trend raises important concerns regarding care coordination. As medical oncologists do not typically perform cystoscopies, coordinating intravesical instillations and surveillance cystoscopies may introduce logistical challenges, particularly when care is delivered across different providers or institutions. Such fragmentation may impact adherence to recommended follow-up protocols. On the other hand, this evolving pattern may offer potential advantages in the future. Recent studies have suggested that combining systemic pembrolizumab or sasanlimab with BCG (22, 23) may be advantageous. If approved by regulatory agencies, these regimens might be easier to adopt in Brazil, as many patients already receive BCG from clinical oncologists, who are the usual prescribers of immunotherapies.

Our findings highlight a heterogeneous use of intravesical chemotherapy in Brazil, with a substantial proportion of urologists either not prescribing it or referring patients to medical oncologists. This variability likely reflects differences in drug availability, institutional protocols, and professional familiarity. The predominance of Gemcitabine is consistent with international trends, particularly in most Eastern countries, where it is also the most common drug prescribed (24). In China, gemcitabine currently figures as the second most prescribed drug, after epirubicin (24). Its predominance may also be influenced by the limited availability of Mitomycin C in Brazil in recent years.

Regarding treatment sequencing, BCG remains the cornerstone of first-line therapy for HR-NMIBC, in line with current guidelines. However, the distribution of second-line strategies after BCG failure reveals a balanced preference between intravesical chemotherapy and radical cystectomy, mirroring global patterns. A recent study has demonstrated similar results worldwide, with RC and intravesical chemotherapy competing as the most common second-line treatments for a BCG-unresponsive NMIBC (23, 24). According to the study, RC was mostly performed in Romania (67%), Arab Countries (50%), and the USA (27%). Brazilian urologists would indicate RC for 41.5% of these patients. Intravesical chemotherapy as a second-line treatment was most used in China (64%), the USA (53%), Middle Eastern countries (30%), and Germany (27%) (15, 17). In Brazil, intravesical chemotherapy was the preferred second line, being mentioned in 43.1% of situations (Figure-1).

Pembrolizumab is approved in Brazil for BCG-unresponsive high risk NMIBC (HR-NMIBC) with carcinoma in situ. Long-term results of these therapies are limited (25), which might explain the low adoption of pembrolizumab for NMIBC worldwide. As reported by Chakra et al., it is fairly adopted, mainly as a third-line option, by 5%-17% of Europeans, 14% of North Americans, 23% of Chinese, and is not adopted in Arab Countries (24). In Brazil, only 5.9% of urologists mentioned pembrolizumab as an eventually prescribed drug for HR-NMIBC. It is interesting, though, that when asked to draw potential sequential treatment lines, a larger proportion of urologists included pembrolizumab in their armamentarium (Figure-1), even when combined with radiotherapy, which is uncommon (26-28).

For Brazilian urologists, the most promising upcoming therapies in the NMIBC scenario are systemic immunotherapy and GEM-IDRS, followed by BCG and intravesical chemotherapy protocols.

Radical cystectomy was mentioned as a routinely indicated practice by most Brazilian urologists. This contrasts with global trends and recommendations (29). RC is a complex procedure, and it is recommended that surgeons perform at least 10 cases per year to achieve acceptable outcomes (30, 31). However, just a few urologists reported referring their patients for RC or to a more experienced surgeon to perform the procedure. It is important to emphasize that not only the surgeon's experience, but also the institution's experience, is crucial for optimal postoperative care following RC (32). Notably, the majority of urologists reported an extremely low procedure volume in the previous year (zero to five procedures). An adequate number of procedures, established at more than 10 per year, was reported by less than 5% of the Brazilian urologists. These findings suggest a possible lack of centralization of complex oncologic procedures in Brazil, which contrast with international recommendations advocating concentration of radical cystectomy in experienced centers. However, it is important to emphasize that our study did not evaluate surgical outcomes such as complications or mortality. Therefore, any potential impact on patient outcomes should be interpreted cautiously and is inferred from previous studies demonstrating the association between higher surgical volume and improved postoperative outcomes (33, 34). Structural barriers, referral pathways and healthcare organizations may contribute to this pattern and warrant further investigation.

This study has several notable strengths. To the best of our knowledge, it is the largest and most comprehensive contemporary study to characterize real-world treatment patterns for both NMIBC and MIBC in Brazil, encompassing a broad national sample and reflecting current clinical practice in the context of recent therapeutic advances. Beyond describing practice patterns, it provides a comprehensive overview of current challenges in bladder cancer care and offers relevant insights for educational initiatives, public health strategies, industry engagement, and the identification of unmet clinical needs. It may also inform the design of future clinical trials by improving understanding of how patients with bladder cancer are managed across the country.

Importantly, the findings highlight substantial variability in clinical practice as well as a concerning mismatch between resource availability and treatment delivery. Despite a response rate of approximately 14%, inherent to survey-based methodologies, the study includes a large absolute sample (n=914) with broad geographic representation across 25 Brazilian federative units. This extensive participation enhances the representativeness of the data and strengthens the validity of the observed patterns. Although respondents were explicitly instructed to base their answers on practice within the supplementary healthcare system, potential contamination from public healthcare (SUS) practices should be acknowledged. Given that many participants work across both systems, some responses may reflect overlapping experiences, potentially influencing the reported patterns of care. In addition, selection bias may be present, as the sample was composed of members of the Brazilian Society of Urology and participants in the Brazilian Congress of Urology, potentially reflecting a more academically engaged population. Furthermore, as with all survey-based studies, the findings are subject to self-report and recall bias. Responses may be affected by memory limitations as well as social desirability bias, potentially leading to overestimation of guideline-adherent practices compared to actual clinical behavior.

Future studies incorporating objective clinical data, registry-based analyses, or observational audits will be important to validate and expand upon these findings.

## CONCLUSIONS

The management of NMIBC in Brazil is characterized by considerable variability, despite general alignment with guideline-based recommendations. Our findings highlight important structural and organizational gaps that influence care delivery, including limited access to intravesical BCG, frequent referral to other specialists for its administration, and restricted availability of office-based flexible cystoscopy.

Together, these factors suggest that barriers related to resource distribution, healthcare organization, and workflow integration may impact both treatment implementation and surveillance strategies. Addressing these challenges represents an important opportunity to educational, organizational, and policy-level interventions aimed at improving the quality and consistency of bladder cancer care in Brazil.

## Data Availability

The original data analyzed during this study is included within this article. Data sharing is not applicable to this article

## ABBREVIATIONS

BCG: Bacillus Calmette-Guérin
SUS: Universal public health care system (Sistema Único de Saúde)
NMIBC: non-muscle invasive bladder cancer
MIBC: Muscle-invasive bladder cancer
TURBT: Transurethral resection of bladder tumors
Re-TURBT: Repeat Transurethral resection of bladder tumors
CFM: Federal Council of Medicine
NBI: Narrow-band imaging
RC: Radical cystectomy
HR-NMIBC: High risk non-muscle invasive bladder cancer
MRI: Magnetic resonance imaging
PET-FDG: Fluorodeoxyglucose-positron emission tomography
EAU: European Association of Urology
AUA: American Urologic Association
NCCN: National Comprehensive Cancer Network
QCI/QPI: Quality Control Indicators / Quality Performance Indicators
SWOG: Southwest Oncology Group
CEP/CONEP: Research Ethics Committee/National Research Ethics Commission (Brazil)
